# Music, mental health, and immunity

**DOI:** 10.1016/j.bbih.2021.100374

**Published:** 2021-10-21

**Authors:** Lavinia Rebecchini

**Affiliations:** Department of Psychological Medicine, Institute of Psychiatry, Psychology & Neuroscience, King's College London, London, UK

**Keywords:** Music, Music therapy, Interventions, Mental health, Immune system

## Abstract

Music is a crucial element of everyday life and plays a central role in all human cultures: it is omnipresent and is listened to and played by persons of all ages, races, and ethnic backgrounds. But music is not simply entertainment: scientific research has shown that it can influence physiological processes that enhance physical and mental wellbeing. Consequently, it can have critical adaptive functions. Studies on patients diagnosed with mental disorders have shown a visible improvement in their mental health after interventions using music as primary tool. Other studies have demonstrated the benefits of music, including improved heart rate, motor skills, brain stimulation, and immune system enhancement. Mental and physical illnesses can be costly in terms of medications and psychological care, and music can offer a less expansive addition to an individual's treatment regimen. Interventions using music offers music-based activities in both a therapeutic environment (Music therapy) with the support of a trained professional, and non-therapeutic setting, providing an atmosphere that is positive, supportive, and proactive while learning non-invasive techniques to treat symptoms associated with various disorders – and possibly modulate the immune system.

## 1 Introduction

Music can play a crucial role to support people at all stages of life: from helping new-born babies develop healthy bonds with their parents to offering vital, sensitive, and compassionate palliative care at the end of life. Singing to new-borns, a widespread activity practised worldwide, has been demonstrated to have valuable benefits such as improving mother-infant interaction and reducing infant distress (Vlismas et al., 2013; Mualem and Klein, 2013a). In the same way, music has been reported as an aid in the reduction of anxiety and agitation in older adults with senile dementia (Sung et al., 2012).

The clinical and evidence-informed use of music interventions to accomplish individualised goals within a therapeutic relationship is defined as Music therapy (Press Release on Mus, 2014). Established as a profession after World War II, Music therapy has become an important part of internationally therapeutic and healthcare settings (Greenberg and Rentfrow, 2017). Even long before that, Pythagoras (c.570 – c.495 BC), the Ancient Greek philosopher and mathematician, prescribed various musical scales and modes to cure an array of physical and psychological conditions (Greenberg and Rentfrow, 2017). Music therapy is part of the Creative Arts Therapies (Mind [Internet]. [cited 2, 2021), in which arts-based activities are used in a therapeutic environment, with the support of a trained professional. Creative Arts Therapies are particularly effective for people who face barriers in expressing themselves with spoken languages, such as individuals with communication deficits or people with mental health difficulties who find it difficult to talk about their experiences and feelings in words. These therapies provide a safe and supportive environment to enable and encourage the patients to express themselves in whatever way possible, encouraging self-expression and development supported by the therapeutic relationship (Ahessy, 2013). Music therapy interventions involve a therapeutic process developed between the patient (or client) and therapist through the use of personally tailored music experiences (de Witte et al., 2019).

This distinguishes Music therapy from other music interventions, offered mainly by medical or healthcare professionals (de Witte et al., 2019; Agres et al., 2021). In fact, music can be utilized not only through a setting lead by a professional Music therapist, but also with individuals and groups in a variety of settings. A wide range of musical styles and instruments can be used, including the voice, enabling people to create their unique musical language to explore and connect with the world and express themselves. Bringing out emotions and thoughts through methods of verbal and nonverbal expression and exploration - such as dance and body movement, music, art (Havsteen-Franklin), and expressive writing (Pennebaker and Chung, 2007; Rebecchini, 2019) - may deactivate the avoidance mechanism and enable the elaboration of emotions and distress. As Juslin and Vastfjall (2008), and Levitin (British Association for M, 2021) have underlined, music has evolved from emotional communication, and the musical components of speech provide honest communication about emotions. Because musical participation and response do not depend solely on the ability to speak, music is particularly effective for people who have difficulty communicating verbally (British Association for M, 2021). Hence, working with music can be life-changing for people affected by disability, injury, or mental disorders.

The potential of music to affect mood, cognition, and behavior has been demonstrated in several studies. On a negative side, some studies have shown that men who were exposed to music with misogynistic lyrics displayed higher levels of aggressive behavior than did those who were exposed to neutral music, especially when the aggressive behavior was directed at a female target person. Men also recalled more negative attributes of women after exposure to misogynistic music (Barongan, Hall). And when the music contained men-hating lyrics, women recalled more negative than positive attributes about men (Fischer and Greitemeyer, 2006). Furthermore, playing loud music incessantly to prisoners has been reported as a form of “music torture” designed to cause extreme discomfort. In fact, it's been a practice against which the legal charity Reprieve set up its “zero-dB” campaign (zero, 2021) the 60th anniversary of the Universal Declaration of Human Rights in December 2008.

There is a vast body of evidence demonstrating that Music therapy is beneficial both physically and mentally. Recently the attention has also focused on whether general music activities, not led by therapists, can enhance the mental health and wellbeing of service users (de Witte et al., 2019; Fancourt et al., 2016). Studies on patients diagnosed with mental disorders such as anxiety, depression, and schizophrenia have shown a visible improvement in their mental health after general music and Music therapy interventions (Fancourt et al., 2016; McCaffrey et al., 2011; Mössler et al., 2011; Erkkilä et al., 2011). Moreover, studies have demonstrated other benefits of music and Music therapy, including improved heart rate, motor skills, stimulation of the brain (Bradt et al., 2013; Magee et al., 2017; Norton et al., 2009) and enhancement of the immune system (Taylor, 1997; Fancourt et al., 2014; Li et al., 2021).

Although music might have initially evolved as a pure art expression with entertainment scopes, it is now clear that music can affect physiological processes, improving physical and mental wellbeing. Consequently, it can have critical adaptive functions.

### 1.1 The role of music since first interactions

The use of the voice through singing is a unique form of interaction and expression. Singing is closely linked to the first forms of interaction between a mother and her infant. The body of research on parent-infant communication has shown that humans' earliest contact has many musical qualities (Trevarthen and Malloch, 2000; Stern, 2010). As Dissanayake suggested (Dissanayake, 2000), a mother's use of rhythmical movement appears to be an essential component for the expression communicated while singing with her infant.

Evidence has underlined that a mother's touch and rhythmical movements, co-created with her infant during musical interactions, are central to the infant's feelings of pleasure (Longhi, 2008) and a healthy mother-infant relationship (Hatch and Maietta, 1991). As a mother emotionally engages with her infant, her sensitivity and affection are communicated through her voice (Fernald, 1989, 1992; Rock et al., 1999), touch and facial expressions (Papoušek and Papoušek, 1987; Stack and Muir, 1992), and rhythmical movements (Hatch and Maietta, 1991). This co-created communicative interaction has been demonstrating a ‘communicative musicality’ due to its intrinsic music and dance-like qualities of the regularity of pulse and sensitive exchange of gestural narratives (Malloch and Trevarthen, 2009). The positive emotional arousal and synchronisation between a mother and her child could be the root of a positive mother-infant relationship, thus essential for future child development (Hodges, 1980; Mualem and Klein, 2013b).

A study conducted by Vlismas et al. (2013) on the effect of music and movement on mother-infant interactions showed that maternal engagement in a music and movement programme resulted in changes to both mothers' and infants' behavior. Specifically, it showed that the effect of the programme increased the mothers' self-reported use of music and enjoyment of interactions with their infants; the mothers' self-reported attachment to their infants; the dyadic reciprocity between mother and infant; and the attentional and affective aspects of mothers' speech.

## 2 Music, music therapy and mental health

Utilising music as a structured intervention in treating mental illnesses such as anxiety, depression and schizophrenia has been reported as beneficial in relieving symptoms (Mössler et al., 2011; Erkkilä et al., 2011), while improving mood and social interactions (Edwards, 2006). Some people with mental disorders may be too disturbed to use verbal language alone efficiently as a therapeutic medium. Thus, the musical interaction might support and provide musical resources and competencies very beneficial for patient's everyday life. Music can have unique motivating, relationship-building, and emotionally expressive qualities (Solli, 2008; Rolvsjord, 2001).

Numerous studies have focused on the effect of music interventions on individuals in clinical settings. Many of these studies concluded that music interventions positively impact mood and anxious or depressive symptoms in both children (Kim and Stegemann, 2016; Yinger and Gooding, 2015; Kemper and Danhauer, 2005) and adults (Carr et al., 2013; van der Wal-Huisman et al., 2018). Reviews of the evidence have suggested that Music therapy may improve mental health in children and adolescents and communication in children with autistic spectrum disorder (Gold et al., 2007; Whipple, 2004). In the same way, clinical reports and pre-experimental studies have suggested that Music therapy may be an effective intervention for adult patients with mental health problems across the world. A recent review which aimed to identify, summarise, and synthesise different experimental studies addressing the effects of Music therapy alone or Music therapy added to standard care on mental health (Lee and Thyer, 2013) has shown the therapy alone or added to standard care to have significantly better effects than psychotherapy (Castillo-Pérez et al., 2010), verbal relaxation (Lin et al., 2011), standard care (Erkkilä et al., 2011; Lin et al., 2011; Yang et al., 2009) and no treatment (Mohammadi et al., 2011; Siedliecki and Good, 2006).

Mental health diseases such as depression and anxiety can have devastating consequences both for patients and their families. Symptoms can be severe and debilitating, leaving individuals alone and isolated. Relationships among family and friends may suffer, and individuals may not receive the support needed to manage their disease. Music can improve symptoms associated with mental illness, but it can also provide an environment for social interaction. As Choi, Lee, and Lim described (Choi et al., 2008), Music therapy helps the individual to express emotions while producing a state of mental relaxation, and consequently it can be beneficial in decreasing symptoms of depression and anxiety, while enhancing interpersonal relationships.

Other music interventions - not lead by a professional music therapist - such as group drumming have been very effective, leading to the enhancement of psychological states, specifically fewer depressive symptoms and greater social resilience (Fancourt et al., 2016): there is a growing body of evidence demonstrating the effects of community group on mental health (Estevao et al., 2021; Clift and Morrison, 2011; Coulton et al., 2015). For example, a study conducted with mothers suffering from postnatal depression found that mothers with moderate-severe depressive symptoms who participated in 10 weeks of music and singing classes with their babies had a significantly faster improvement in symptoms than mothers who participated in usual care groups (Fancourt and Perkins, 2018).

In the same way, using Music therapy to decrease psychological stress during pregnancy has been reported as an appropriate alternative therapy for pregnant women suffering from mental health problems attempting to avoid the side effects associated with medication. A study conducted in 2007 with the aim of examining the effects of Music therapy on reducing psychological stress during pregnancy reported that listening to music for at least 30 minutes daily substantially reduced psychological stress, anxiety, and depression (Chang et al., 2008). Hence, listening to music daily during pregnancy can generate considerable health benefits.

These experimental results indicate that music promotes psychological health both during pregnancy, and the entire lifetime; it can be easily used in many environments, and it can also be tailored to personal preferences to enhance mental health.

### 2.1 Music and immune system

The immune system, composed by molecular and cellular components, is a complex system of structures and processes that have evolved to protect us from disease. The function of these components is divided up into nonspecific mechanisms, those which are innate to an organism, and responsive responses, which are adaptive to specific pathogens. The innate immune system represents the first line of defense against infection and includes cells and proteins that are nonspecific to particular antigens. The adaptive immune system provides a secondary, antigen-specific response during which cells with a memory for specific pathogens are created. The adaptive immune system has the capacity to recognize and respond to virtually any protein or carbohydrate imaginable; yet, without the innate immune system to instruct it—in effect, telling it whether, when, how, and where to respond—it is powerless (Clark and Kupper, 2005). As the literature shows, the immune system is strongly associated with mood, psychological condition, and hormonal balance (Segerstrom and Miller, 2004). Thus, as a result of negative mood, psychological stress affects the immune system and may cause dysregulation leads to a change in the humoral and cellular immunity and increases health risks.

Psychological stress can have detrimental effects on both immune system responses, leading to a weakening of defenses against new pathogens and increasing in systemic inflammation (Chanda and Levitin, 2013a; Maddock and Pariante, 2001). While inflammation is a local, protective response to microbial invasion or injury, it must be fine-tuned and regulated precisely, because deficiencies or excesses of the inflammatory response cause morbidity and shorten lifespan (Tracey, 2002; Bassi et al., 2018). Because stress can be a predisposing factor to diseases associated with immunologic responses (Maddock and Pariante, 2001), increased exposure to stressful situations expands the risk of mental and physical disorders (Hazelgrove et al., 2021). Acute stress can affect basal sensitivity, increasing or decreasing pain threshold in acute and chronic pain processes. For the fact that acute and chronic pain are potent stressors, they can alter the body homeostasis: pain can be an activator of the hypothalamic-pituitary-adrenal axis (HPA axis), the major system responsible for stress responses, which may be hypoactive or hyperactive under chronic or persistent stress conditions (Timmers et al., 2019). In turn, the HPA axis modulation directly affects the release levels of glucocorticoids, ‘hormones of stress’, which induce anti-inflammatory and immunosuppressive effects at pharmacological doses, whereas at physiological levels they play an essential regulatory role in the immune system (Pariante and Miller, 2001). Thus, stress can negatively affect the cardiovascular, neuroendocrine, and immune systems, which, consequently, may impair recovery, increase the risk for adverse effects, and delay hospital discharge (Biondi and Zannino, 1997).

Although psychological stress cannot be eliminated, there are ways in which the perception of stress and ability to adapt to stressors can be altered: music has been adapted as a form of stress management and studies have confirmed the effect of music on the reduction of stress responses in the cardiovascular and endocrine system (Taylor, 1997; Mojtabavi et al., 2020). Specifically, music has been shown to modify heart rate, respiration rate, perspiration, and other autonomic systems (Blood et al., 1999), supporting reports that many people use music to achieve physical and psychological balance. Lifestyle choices that reduce stress are thought to be highly protective against diseases (Dimsdale, 2008), and music may be among these (Dileo et al., 2007; Nilsson, 2008).

The human's biological stress response is highly adaptive in the short term: it is an *elegant choreography* (Chanda and Levitin, 2013b) of neuroendocrine, autonomic, metabolic, and immune system activity that involves multiple feedback loops at the level of the central and peripheral nervous systems (Landgraf and Neumann, 2004). Together these systems trigger short term adaptive behaviours, including arousal, vigilance, focused attention, and temporarily inhibit functions that are nonessential during a crisis, such as eating, digestion, growth, and sex drive. At the same time, cardiovascular changes such as elevated heart rate and rapid breathing are helpful to increase oxygenation and glucose supply to the brain and skeletal muscles.

However, as already mentioned, the prolonged activation of these systems has devastating consequences for health. Continuous and elevated circulating levels of glucocorticoids (e.g., cortisol) act as neurotoxins, weakening the ability of neurons and other cells to resist injury and making them more vulnerable to the effects of toxins and the normal attrition process (Landgraf and Neumann, 2004). Furthermore, although glucocorticoids act as an immunosuppressant under acute stress conditions, they may promote a state of chronic low-grade inflammation in the long term (Gouin et al., 2008). These neurotoxic and pro-inflammatory effects of chronic stress have been linked to a host of adverse health outcomes such as susceptibility to infectious diseases, anxiety and depression (Pitharouli et al., 2021), and cardiovascular diseases (Chrousos, 2009; Lupien et al., 2009).

As many studies have demonstrated, neuroinflammation is the cause of several mental diseases such as depression and anxiety (Zheng et al., 2021; Troubat et al., 2021). Hence, attention has increasingly focused on the effect of music as a possible anti-inflammatory mechanism in these central inflammatory conditions. A recent work conducted by Dasy Fancourt (2014) - the first systematic review that aimed to assess published studies dealing with psychoneuroimmunological effects of music - showed that music can have effects on various neurotransmitters, cytokines, and hormones (Fancourt et al., 2014). Specifically, fifty-six of the sixty-three studies included in the author's systematic review linked psychoneuroimmunological effects of music to the stress response.

Salivary Immunoglobulin A (s-IgA), a first-line in the defence against bacterial and viral infections (Woof and Kerr, 2006) and a reliable marker of the functional status of the entire mucosal immune system (Hucklebridge et al., 2000), has been revealed to be particularly responsive to music, increasing following exposure to a range of styles of music including both relaxing and stimulating music, as well as for both active involvements and simply listening to recorded music (Fancourt et al., 2014). Strong patterns have also been noticed concerning cortisol, which repeatedly decreased in response to relaxing recorded music (Fancourt et al., 2014). There also appeared to be patterns in the response of epinephrine and norepinephrine, which have been shown to decrease in response to relaxing recorded music (Leardi et al., 2007).

Another study conducted to determine if (i) musical activity could produce a significant change in the immune system measured by s-IgA, and if (ii) active participation in musical activity had a different effect on the immune system than passive participation showed that S-IgA levels of the active group (playing music and singing) had more significant increase than those of the passive group (listening only) (Kuhn, 2002). This result suggested that active participation in musical activity produces a more significant effect on the immune system than passive participation.

Overall, changes have been observed across various immune response biomarkers, including leukocytes, cytokines, immunoglobulins, and hormones and neurotransmitters associated with immune response (Fancourt et al., 2014). Music has begun to be taken seriously in healthcare settings as research findings have started to link the beneficial effects of music on stress to a broader impact on health (Haake, 2011). If music can mediate anti-inflammatory effects, evidenced by decreased levels of inflammatory biomarkers (see Table 1), there may be biological plausibility for its use in the care of ill patients. The results of these studies provide further confirmation that the immune system can be enhanced by music and, as Daisy Fancourt has underlined, the trend towards positive findings of the effect of music on psychoneuroimmunological response strongly supports further investigation in this field (Fancourt et al., 2014).

**Table 1:** *Markers of inflammation and immune response influenced by music*.

Type of music activities	Peripheral markers	Brain regions
Listening to relaxing/pleasant music (Fancourt et al., 2014; Leardi et al., 2007; Brown et al., 2004; Jeffries et al., 2003; Koelsch et al., 2006)	Increased levels of: - salivary Immunoglobulin A (s-IgA) Decreased levels of: - cortisol - epinephrine - norepinephrine	Activation of: - ventral striatum: nucleus accumbens (NAc) - opioid-rich midbrain nuclei - anterior superior insula - Rolandi operculum Deactivation of: - amygdala - hippocampus - parahippocampal gyrus - temporal poles^f^
Listening to techno music (Gerra et al., 1998)	Increased levels of: - plasma cortisol - adrenocorticotropic hormone - prolactin - growth hormone - norepinephrine levels	–
Active participation in musical activity (playing, singing) (Kuhn, 2002; Jeffries et al., 2003)	Increased level of: - salivary Immunoglobulin A (s-IgA)	Activation of: - ventral striatum: nucleus accumbens (NAc)

#### 2.1.1 Other biological effects

In the last decade, there has been growing interest in music's chemical and biological effects (Table 1) (Khan et al., 2018). Some studies have focused on whether music can affect the same neurochemical reward systems as other reinforcing stimuli. Does music have the earmarks of a rewarding stimulus, including the ability to motivate an individual to learn and engage in goal-directed behavior to obtain a pleasurable feeling (Chanda and Levitin, 2013b)? As Salimpoor et al. have underlined (Salimpoor et al., 2015), dopamine activity can explain why an individual would be motivated to keep listening to a piece of music, or to seek out that music in the future. However, it cannot alone explain the experience of pleasure when listening to music. Berridge and colleagues described ‘hedonic hotspots’ in the nucleus accumbens (NAc) and ventral pallidum that are explicitly linked to the display of pleasure and are triggered by opioid signalling (Berridge and Kringelbach, 2013). Thus, there are crucial interactions between the dopamine and opioid systems. A rapid increase in dopamine release in humans induces euphoria, with the level of euphoria correlating with the level of ventral striatal dopamine release, which also leads to robust increases of endorphin release in the NAc (Drevets et al., 2001). On the other hand, opioid antagonists block the subjective ‘high’ caused by strong dopamine release (Jayaram-Lindström et al., 2004). Consequently, it seems reasonable to hypothesize that a strong induction of dopamine release caused by music can trigger opioid stimulation of so-called hedonic hotspots. In the other direction, the opioid system robustly modulates dopamine release in to the NAc (Hjelmstad et al., 2013). This likely provides a mechanism through which music that is experienced as pleasing can enhance dopamine-mediated positive prediction error signaling and reinforcement learning. Thus, the association of dopamine release and NAc activation during peak musical pleasure may be a direct manifestation of this opioid–dopamine interaction (Salimpoor et al., 2015).

There is an increasing body of evidence demonstrating the functional activation (Blood and Zatorre, 2001; Brown et al., 2004; Jeffries et al., 2003; Koelsch et al., 2006), network connectivity (Menon and Levitin, 2005), and central dopamine release (Salimpoor et al., 2011) during the perception of pleasurable music. A review conducted by Chanda and Levitin (2013b) showed that studies that used positron emission tomography (PET) to investigate regional cerebral blood flow (rCBF) during experienced musical pleasure (Blood and Zatorre, 2001; Brown et al., 2004; Jeffries et al., 2003) suggested that music reward involve the activation of the NAc, as well as opioid-rich midbrain nuclei known to regulate morphine analgesia and descending inhibition of pain (Jeffries et al., 2003). NAc activation was also reported during listening to unfamiliar pleasant music compared to rest (Brown et al., 2004) and during singing compared to speech (Jeffries et al., 2003). On the other hand, listening to techno-music induced changes in neurotransmitters, peptides and hormonal reactions, related to mental state and emotional involvement: techno music increased plasma cortisol, adrenocorticotropic hormone, prolactin, growth hormone and norepinephrine levels (Gerra et al., 1998). The neuroendocrine pattern induced by this fast music (techno music) turned out to be similar to the biological reaction to psychological stress (Henry, 1992).

Other studies that used higher resolution functional magnetic resonance imaging (fMRI) to investigate the neural correlates of music pleasure (Koelsch et al., 2006; Menon and Levitin, 2005; Salimpoor et al., 2011; Janata, 2009) showed that musical reward is dependent on dopaminergic neurotransmission within a similar neural network as other reinforcing stimuli: pleasant (consonant – positive emotional valence) and unpleasant (dissonant – negative emotional valence) music were contrasted, and the results confirmed activation of the ventral striatum and Rolandi operculum during pleasurable music listening, while strong deactivations were observed in the amygdala, hippocampus, parahippocampal gyrus, and the temporal poles in response to pleasant music (Koelsch et al., 2006). Activation of the anterior superior insula in response to pleasant music has also been observed: a significant finding because of the insula's connectivity to the NAc and its role in the activation of the emotional circuitry and reward system (Pavuluri et al., 2017) which, in turn, increases the innate and adaptive immune system (Ben-Shaanan et al., 2016). All these structures have previously been implicated in the emotional processing of stimuli with (negative) emotional valence (Heinzel et al., 2005; Siegle et al., 2002). The results of the studies mentioned above indicate that these structures respond to auditory information with emotional valence, and that listening to music has the capacity to up-as well as down-regulate neuronal activity in these structures.

## 3 Conclusion and limitations

The increasing evidence of the benefits of music activities and Music therapy provided by the literature is a driving force for developing music-based therapies services in the health care sector. By promoting physical and psychological health, music can be an effective treatment option suitable for every environment and people of every age, race, and ethnic background.

Since music is a complex topic, there are some aspects that this mini review has not fully addressed, such as the role of the autonomic nervous system involved in musical activities; the involvement of music as a possible component of an “enriched environment” (Kempermann, 2019); and, finally, the beneficial effects of rhythmical movements and physical musical activities, and their contribution to the preference for treatment options.

Figure 1. Lavinia Rebecchini is an Italian psychologist currently doing a Ph.D at the Department of Psychological Medicine at the Institute of Psychiatry, Psychology and Neuroscience (IoPPN), King's College London. She graduated from Università Cattolica del Sacro Cuore of Milan and, after completing her Master of Science in Developmental Psychology with full marks, she decided to move to London to broaden her horizons. She started as an intern at the Perinatal Psychiatry section of the Stress, Psychiatry, and Immunology Laboratory (SPI Lab) at the IoPPN and, after being hired as a Research Assistant, she then decided to further cultivate her strong interest in the perinatal mental health field with a PhD. She has always been interested in perinatal psychiatry and the relationship between mothers and their children. Her Ph.D at the SPI Lab is concentrating on mother-infant interaction with mothers suffering from perinatal depression. With her Ph.D project, she focuses on which implications perinatal depression may carry for the developing mother-infant relationship. She looks at whether an intervention of music and singing sessions can help mothers develop compensatory skills to interact with their children appropriately so to better respond to their infants' needs. In addition to her academic experiences, during her free time, she has always volunteered to help children and families in need. She is determined and enthusiastic, and her eight years' experience in alpine skiing competitions has allowed her to build strong determination in achieving her goals.

## Declaration of competing interest

The author Lavinia Rebecchini declares that there are no conflicts of interest.
